# Fundamentals of Deep‐Subwavelength Near‐Field Imaging With Hyperbolic Polaritons

**DOI:** 10.1002/nap2.70089

**Published:** 2026-04-17

**Authors:** Tianran Yu, Zhenyang Cui, Sihao Xia, Zhiwei He, Huaping Wang, Lian Shen, Hongsheng Chen, Yingjie Wu

**Affiliations:** ^1^ State Key Laboratory of Extreme Photonics and Instrumentation, ZJU‐Hangzhou Global Scientific and Technological Innovation Center Zhejiang University Hangzhou China; ^2^ The Electromagnetics Academy at Zhejiang University College of Information Science and Electronic Engineering Zhejiang University Hangzhou China; ^3^ Zhejiang Key Laboratory of Intelligent Electromagnetic Control and Advanced Electronic Integration Jinhua Institute of Zhejiang University Zhejiang University Jinhua China

**Keywords:** deep‐subwavelength resolution, hyperbolic polariton beams, interface polaritons, near‐field imaging, negative refraction

## Abstract

Interface polaritons with extreme electromagnetic confinement enable near‐field imaging with resolution far beyond the diffraction limit. Natural polaritonic materials supporting low‐loss and anisotropic polariton modes have emerged as versatile platforms for deep‐subwavelength imaging, yet the fundamental limits of their resolving power remain unexplored. Here, we uncover the intrinsic resolution limit of polaritonic imaging by linking it to the linewidth of hyperbolic polariton beams, which originates from the material's optical properties and scales linearly with thickness. By exploiting negative refraction in vertically stacked heterostructures, we further propose a strategy to achieve scalar fidelity under proper thickness configurations and reveal a potential pathway to mitigating geometrical distortion in polaritonic imaging. Our findings elucidate the fundamental physics of near‐field imaging with hyperbolic polaritons, providing guiding principles for improving both its scalar and geometrical fidelity toward practical applications.

## Graphical abstract

Hyperbolic polaritons enable deep‐subwavelength near‐field imaging. This work reveals the intrinsic resolution limit of polaritonic imaging by linking it to the linewidth of hyperbolic polariton beams. A negative‐refraction‐based strategy is proposed to achieve scale fidelity under appropriate thickness configurations and to mitigate geometrical distortion through optimal structural design, laying the foundation for imaging beyond the diffraction limit.

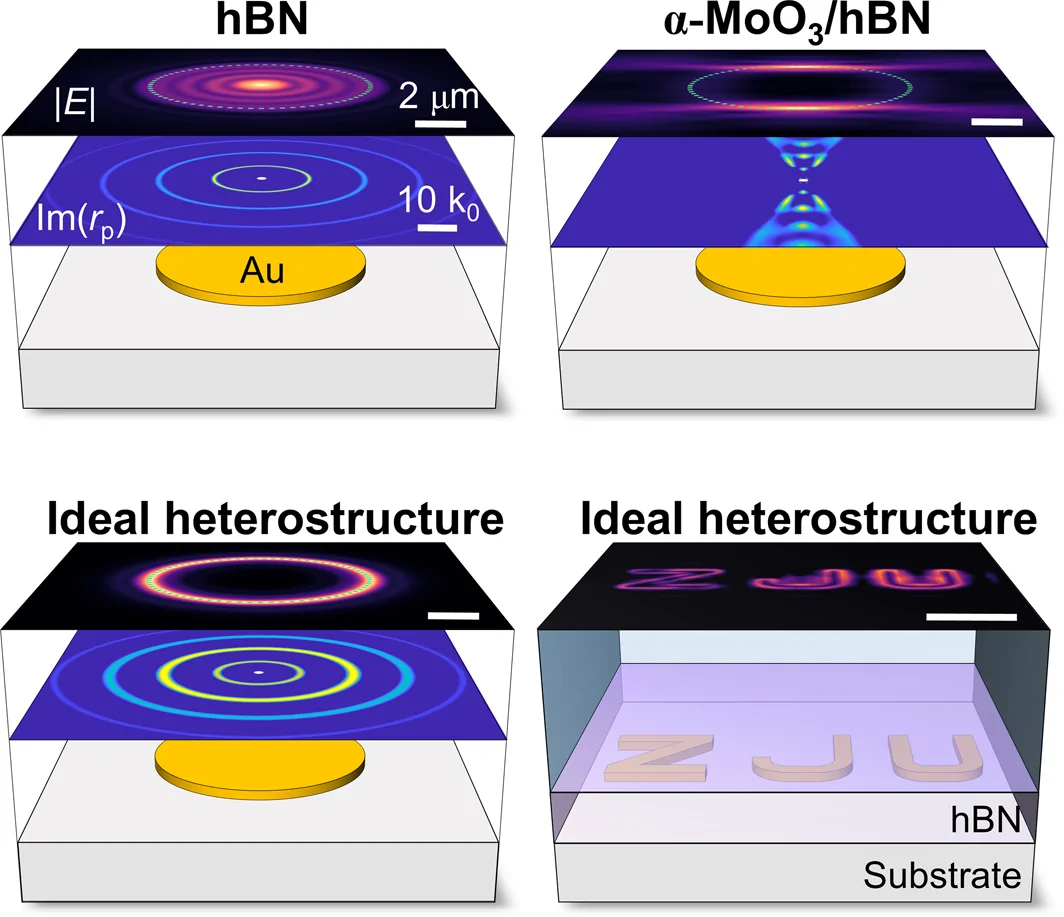

## 1 Introduction

The continuing pursuit of high‐resolution imaging beyond the diffraction limit has stimulated tremendous efforts in developing novel imaging mechanisms and optical devices. For instance, superlenses with negative refractive indices can overcome the diffraction limit by amplifying evanescent waves [1, 2, 3, 4], while hyperlenses exploit hyperbolic dispersions to access even larger wavevectors and achieve higher resolutions [5, 6, 7]. Metalenses composed of laterally arranged subwavelength elements, known as metasurfaces, have enabled additional functionalities and tunability [8, 9, 10]. Among these approaches, interface polaritons have emerged as a promising near‐field platform [11, 12, 13], owing to their strong electromagnetic confinement and exceptionally high in‐plane momenta [14]. However, the need for elaborate nanofabrication and the associated optical losses hinders their practical implementation.

The development of natural hyperbolic materials [15, 16, 17, 18, 19], such as hexagonal boron nitride (hBN) and α‐phase molybdenum trioxide (α‐MoO_3_), has opened new opportunities for low‐loss near‐field focusing and imaging [20, 21, 22]. Due to their unique lattice structures, these materials sustain low‐loss and anisotropic polaritons, enabling nondestructive super‐resolution imaging [23, 24, 25]. Nevertheless, due to fabrication imperfections and the finite spatial resolution of characterization methods, the ultimate limits of hyperbolic–polariton‐based imaging remain elusive. A comprehensive understanding of the origin, constraints, and optimization strategies for such imaging systems is therefore urgently needed.

In this study, we theoretically reveal the fundamental limits to the resolution and fidelity of deep‐subwavelength imaging mediated by hyperbolic polaritons in natural materials. We establish that the spatial resolution is governed by the linewidths of polariton beams, which scale linearly with their radial propagation distance. Furthermore, we demonstrate that α‐MoO_3_/hBN heterostructures supporting negative refraction allow scalar fidelity at selective frequencies through appropriate thickness design, while optimal configuration design is crucial for improving their geometrical fidelity. Our findings elucidate the physical origin of deep‐subwavelength polaritonic imaging, providing a general framework for evaluating and enhancing its performance.

## 2 Results and Discussion

### 2.1 Linewidths of Hyperbolic Polariton Beams

We begin by examining the radiation characteristics of hyperbolic polaritons within their host medium. Taking hBN as an example, we numerically calculate dipole‐induced hyperbolic field distribution by solving the first kind and first order Hankel function [26, 27], following the Clemmow's scaling method for hyperbolic media [28] (see Supporting Information S1: Note 1 for calculation details). Figure 1a shows the calculated electric field in the *y*−*z* plane of a sufficiently thick h‐BN crystal, where only the upper half is shown for clarity. At a frequency *ω* = 790 cm^−1^, because of the opposite signs of the in‐ and out‐of‐plane dielectric permittivities ($\varepsilon_{\text{hBN}}^{\text{xy}}\cdot \varepsilon_{\text{hBN}}^{z}<0$), polaritons manifest as out‐of‐plane hyperbolic beams. The beam center tilts by an angle *θ* with respect to the *y* direction, defined as [20, 21, 29]

(1) $$\theta =\text{Re}\left[\mathrm{atan}\left(\sqrt{-\frac{\varepsilon_{\text{hBN}}^{z}}{\varepsilon_{\text{hBN}}^{\text{xy}}}}\right)\right].$$

**FIGURE 1:**
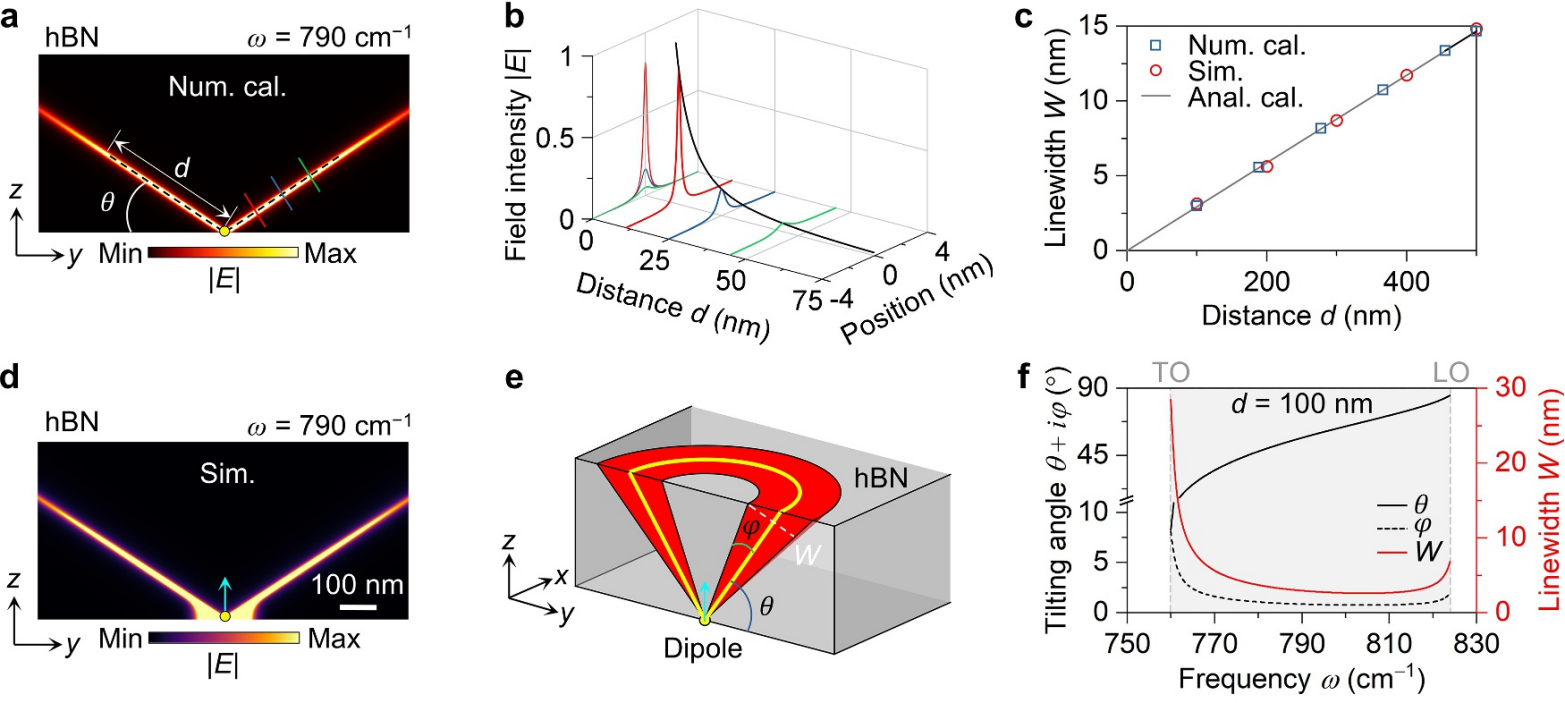
Hyperbolic polariton beams in hBN. (a) Calculated electric field intensity of hyperbolic polariton beams launched by a dipole (yellow circle) in an hBN bulk crystal. The central beam axis tilts by an angle of *θ* to the *y* direction. (b) Field intensity as a function of the radial distance (*d*) from the point source, extracted along part of the black dashed line in (a). Back projection shows three cross‐sectional profiles taken along the beam‐normal direction. (c) Linewidth (*W*) of polariton beams as a function of *d*. (d) Full‐wave simulation under identical conditions to (a). (e) Schematic illustration of hyperbolic polariton beams characterized by a tilting angle *θ* and a divergence angle *φ*. (f) Complexed tilting angle (*θ* + *iφ*) and corresponding *W* as a function of *ω*.

Due to intrinsic absorption loss [30], represented by the imaginary parts of the dielectric permittivities, the beam intensity decays exponentially with the radial distance *d* from the point source (Figure 1b). Simultaneously, the beam linewidth *W*, defined as the full width at half maximum (FWHM) along the beam–normal direction, increases linearly with *d* (Figure 1c, squares).

To validate these results, we performed full‐wave simulations using the finite element method, with a *z*‐polarized point dipole positioned at the center of the hBN crystal (see Methods), which also allows linewidth extraction in more complicated structures (see below). The simulated field distribution (Figure 1d) closely matches the numerical solution in Figure 1a, except for minor interference from higher‐order modes near the dipole (Supporting Information S1: Figure S1). The extracted linewidths (Figure 1c, circles) also exhibit a linear dependence on *d*, in quantitative agreement with the numerical calculations.

To gain a phenomenological understanding of the linear relationship between *W* and *d*, we introduce an analytical model based on the imaginary component of the tilting angle:

(2) $$\varphi =\left|\text{Im}\left[\mathrm{atan}\left(\sqrt{-\frac{\varepsilon_{\text{hBN}}^{z}}{\varepsilon_{\text{hBN}}^{\text{xy}}}}\right)\right]\right|.$$

Because of the complex‐valued dielectric permittivities, the tilting angle α of a polariton beam becomes a complex quantity, α=θ+iφ. While *θ* defines the beam direction, φ represents the absorption‐induced divergence angle, as schematically illustrated in Figure 1e. Geometrically, the beam linewidth can thus be expressed as the following:

(3) W=2dtan(φ).

The analytical result (gray line in Figure 1c) agrees remarkably well with both the numerical calculations and full‐wave simulations. A similar result is also obtained at other frequencies or in the configuration composed of a *y*‐polarized dipole (Supporting Information S1: Figures S2 and S3). This demonstrates that the complex‐valued tilting angle defines both the propagation direction (*θ*) and the divergence (*φ*) of hyperbolic polariton beams within the host medium. We note that this analytical method just focuses on intrinsic polaritons in their host medium and does not include external loss channels. Nevertheless, it provides an efficient method to directly calculate the linewidth of polariton beams without extracting them from electric fields obtained through numerical calculations or full‐wave simulations.

Since the dielectric permittivities of hBN vary with frequency, both *θ* and *φ* are frequency dependent, as shown in Figure 1f. Within the Reststrahlen band (RB)—bounded by the transverse optical (TO) and longitudinal optical (LO) phonon frequencies—*θ* increases monotonically with *ω* and approaches 90° near the LO frequency. In contrast, *φ* peaks at the TO frequency and exhibits a nonmonotonic behavior, leading to a frequency‐dependent linewidth reaches its minimum at 804 cm^−1^.

A similar linear dependence of *W* on *d* is also observed for hyperbolic polaritons supported by α‐MoO_3_ (Supporting Information S1: Figure S4). The slope in that case can likewise be described by Equation (3), confirming the general applicability of our phenomenological model. Although a more rigorous derivation would further illuminate the underlying physics, this model already provides a convenient and predictive framework for estimating the intrinsic linewidths of hyperbolic polariton beams.

### 2.2 Polaritonic Imaging With an Individual Slab

In an hBN slab of finite thickness *h*_hBN_, hyperbolic polariton beams undergo multiple reflections between the top and bottom interfaces. As shown in Figure 2a, for *h*_hBN_ = 300 nm, the strong momentum mismatch at the interface results in total internal reflection, producing evanescent fields that decay exponentially into the surrounding air. These near‐field signals can be detected using techniques such as scanning near‐field optical microscopy (SNOM), with spatial resolution down to tens of nanometers determined by the tip radius [31, 32], thereby enabling super‐resolution imaging mediated by hyperbolic polaritons.

**FIGURE 2:**
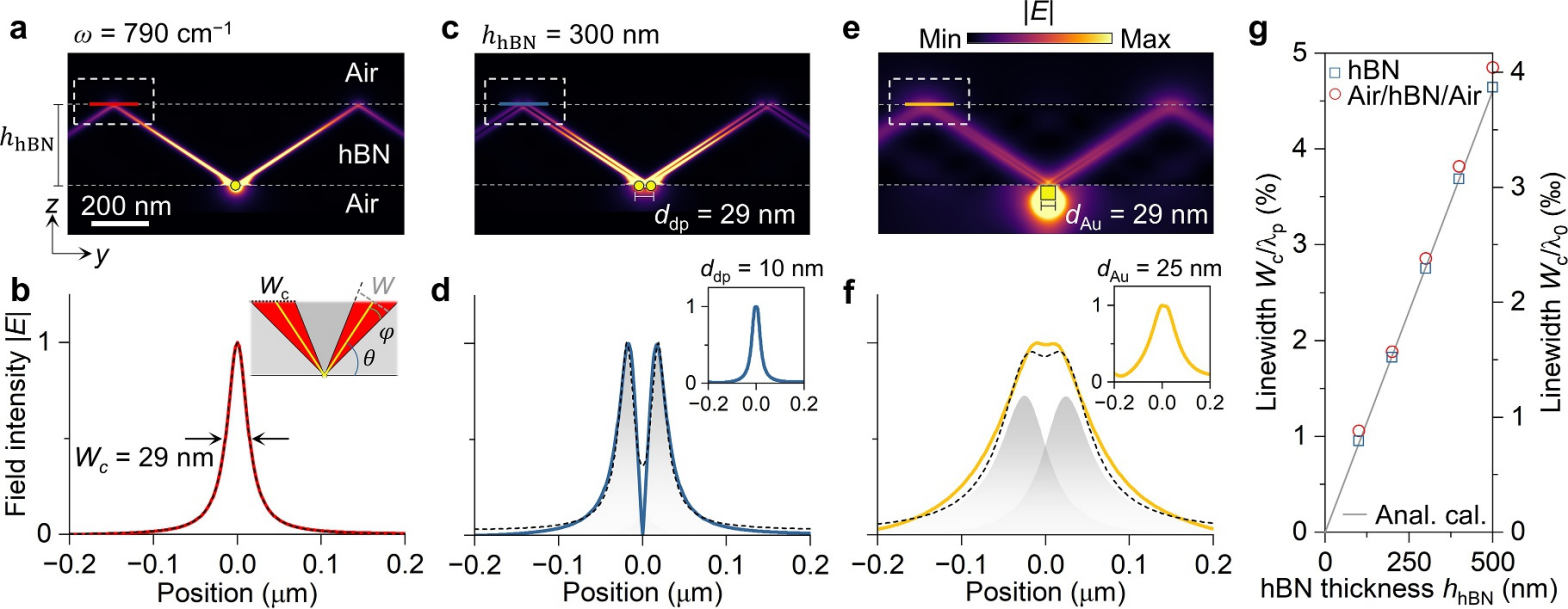
Polaritonic imaging with an individual hBN slab. (a) Simulated field distribution in an hBN slab of thickness *h*_hBN_ = 300 nm. (b) Line‐scan profile (red curve) and asymmetric Lorentzian fitting (black curve) along the red line in (a). (c, d) Same as (a, b) but excited by two laterally positioned dipoles separated by a distance *d*_dp_. Gray areas indicate the two component peaks. Inset is the line‐scan profile with *d*_dp_ = 10 nm, corresponding to the resolution limit defined by the Sparrow criterion. (e, f) Same as (c, d) but with dipole pairs replaced by a gold antenna of length *d*_Au_. (g) Thickness‐dependent cross‐sectional linewidth (*W*_c_) normalized to the polariton wavelength (*λ*_p_, left) and to the free‐space wavelength (*λ*_0_, right).

In typical near‐field imaging experiments, the observation plane corresponds to the top surface of the hBN slab, which is generally not perfectly perpendicular to the polariton propagation direction, except when *θ* ≈ 90°. This slight misalignment requires that the extracted linewidths (*W*_c_) at lateral cross‐sections in Figure 2b fitted by an asymmetric Lorentzian function. According to the schematic in the inset of Figure 2b, *W*_c_ follows

(4) $$W_{c}=h_{\text{hBN}}\left[\cot\left(\theta -\varphi \right)-\cot\left(\theta +\varphi \right)\right].$$

To further assess the imaging performance, we consider two laterally positioned dipoles separated by a distance *d*_dp_. As shown in Figure 2c,d, two distinct peaks emerge on the top surface, corresponding to the projected dipole pair. The determination of resolution limit in this scenario parallels that of the classical diffraction limit in optics. To quantitatively evaluate the resolving power, we employ the well‐known FWHM resolution limit (*R*_p_) defined as the distance between two peaks exactly equal to the polariton linewidth *R*_p_ = *W*_c_. According to Equation (4), this is corresponding to *R*_p_ = 29 nm. An even higher resolution could be claimed if adopting other criteria with relaxed limitations, such as the Sparrow criterion [33], dictated by the existence of the dip between the two peaks. Under this criterion, the resolution limit is estimated to be 10 nm (Figure 2d, inset).

In near‐field experiments, gold antennas are commonly used to launch polaritons. As shown in Figure 2e, a gold antenna with a rectangular cross‐section and length *d*_Au_ is placed beneath the hBN slab. Although two peaks can still be observed in the near‐field signals (Figure 2f), they are less distinguishable than those excited by dipole pairs. This indicates that the gold antenna introduces additional losses into the system, leading to broader linewidths than those predicted by the theoretical model. Additional simulations in Supporting Information S1: Figure S5 show that this effect becomes more pronounced as the antenna length increases. Therefore, the Sparrow criterion may be more appropriate for determining the resolution in this configuration, yielding a value of 25 nm (Figure 2f, inset), which is larger than that obtained for the dipole‐launched configuration.

Unlike conventional optical imaging, where the diffraction limit scales with the free‐space wavelength (*λ*_0_), the resolution in polaritonic imaging does not directly correlate to polariton wavelength (*λ*_p_). Instead, it is determined by the cross‐sectional linewidth of hyperbolic polariton beams. This linewidth is insensitive to the air superstrate and substrate but increases linearly with the slab thickness (Figure 2g). Since practical devices typically employ slabs with thicknesses of several hundred nanometers, polaritonic imaging naturally achieves deep‐subwavelength resolution—well below both the polariton and free‐space wavelengths—reaching 2.7 × 10^−2^ *λ*_p_ or 2.3 × 10^−3^ *λ*_0_ at *h*_hBN_ = 300 nm. Although even higher resolutions can, in principle, be attained with thinner hBN slabs (Supporting Information S1: Figure S6); however, in this regime, the achievable resolution becomes limited by the precision of the experimental instrumentation rather than by the intrinsic properties of the polaritons.

### 2.3 Polaritonic Imaging Based on Negative Refraction

While an individual slab enables deep‐subwavelength polaritonic imaging, accurately identifying object boundaries can be challenging in some cases. For instance, when the antenna length becomes sufficiently large, strong polaritonic resonance arises, producing additional signal maxima within the antenna region, as shown by gray curves in Figure 3a. These peaks obscure the true object edges, complicating direct image interpretation. Although post‐processing algorithms can reconstruct object geometry from composite near‐field images [22], a more direct approach for extracting geometric features is highly desirable.

**FIGURE 3:**
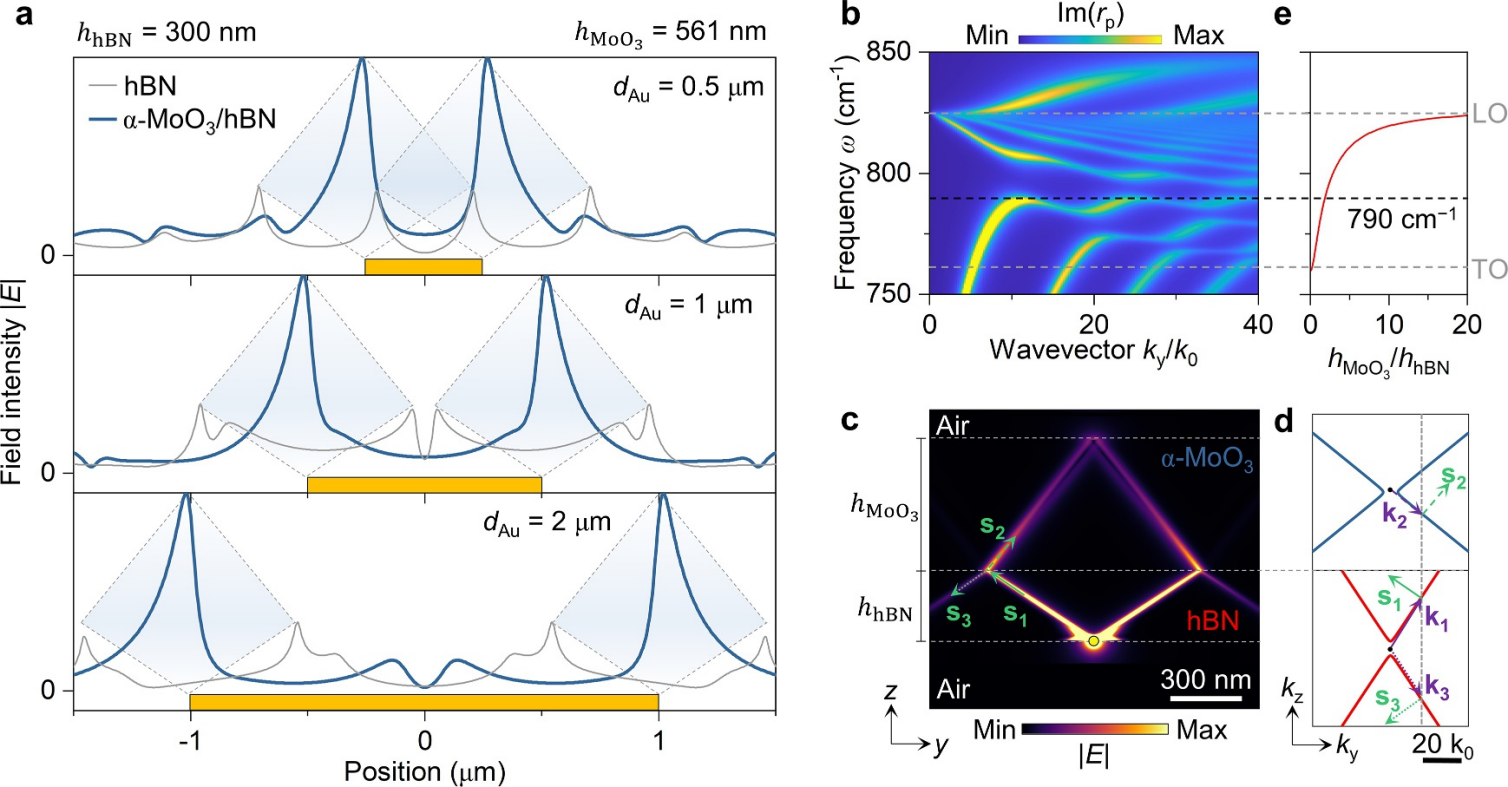
Polaritonic imaging based on negative refraction. (a) Near‐field intensity profiles on the surfaces of an individual hBN slab (gray curves) and an α‐MoO_3_/hBN heterostructure (blue curves) placed on gold antennas with varying widths (*d*_Au_). Diamonds denote the trajectories of negatively refracted polariton beams. For comparison, the intensities over individual hBN slabs are normalized to trajectory waists. (b) Dispersion in the α‐MoO_3_/hBN heterostructure. (c) Simulated field distribution in the α‐MoO_3_/hBN heterostructure excited by a point dipole. (d) Schematic of momentum conservation between α‐MoO_3_ (top) and hBN (bottom) on their respective IFCs. (e) Frequency‐dependent $\frac{h_{{\text{MoO}}_{3}}}{h_{\text{hBN}}}$ for scale‐fidelity polaritonic imaging.

To enhance image fidelity, we introduce a strategy based on the negative refraction of hyperbolic polaritons at the interface of vertically stacked α‐MoO_3_/hBN heterostructures [34, 35, 36, 37, 38]. Within the partially overlapped RBs of α‐MoO_3_ and hBN, strong coupling occurs between these two polariton modes owing to their opposite group velocity directions, leading to multiple anti‐crossings in the hybridized dispersion in Figure 3b.

In this regime, hyperbolic beams emitted from a point dipole beneath the hBN layer bend backward and converge near the top surface of α‐MoO_3_, forming a sharply focused near‐field image (Figure 3c). The simulated beam trajectories are in good agreement with the incident (**S**_1_), refracted (**S**_2_), and reflected (**S**_3_) Poynting vectors, geometrically derived from momentum conservation on the isofrequency contours (IFCs) at the α‐MoO_3_/hBN interface (Figure 3d). The IFCs were calculated separately for the α‐MoO_3_ and hBN layers and do not consider their mutual interaction. As the hyperbolic polariton beams are dominated by high‐wavevector modes, their directions can be sufficiently determined from these hyperbolic IFCs, whose asymptotic lines are governed solely by the dielectric permittivities of the polaritonic materials.

A similar negative refraction behavior is observed for polariton rays launched from the antenna corners, producing near‐field intensity peaks that precisely correspond to the antenna edges. This scale preservation remains robust across a wide range of antenna widths, as shown by the blue curves in Figure 3a. The corresponding field distribution profiles are provided in Supporting Information S1: Figure S7.

In contrast to single‐slab imaging—where scale fidelity is achieved only at specific frequencies satisfying *θ* ≈ 90°—the α‐MoO_3_/hBN heterostructure maintains consistent image scaling across a broad spectral range within the overlapping RBs (specifically, between the TO and LO frequencies of hBN), provided that the thickness ratio satisfies $\frac{h_{{\text{MoO}}_{3}}}{h_{\text{hBN}}}=\frac{\tan\left(\theta_{{\text{MoO}}_{3}}\right)}{\tan\left(\theta_{\text{hBN}}\right)}$, which equals 1.87 at *ω* = 790 cm^−1^ (Figure 3e). Additional results at other frequencies are presented in Supporting Information S1: Figure S8. Consistent with previous results [35], the achievable resolution in such heterostructures continues to scale linearly with the slab thicknesses, which can be routinely explained by the linewidth dependence discussed above (see Supporting Information S1: Note 2 and Figure S9 for linewidth analysis in heterostructures). Furthermore, interchanging the order of α‐MoO_3_ and hBN layers yields a comparable focusing effect (Supporting Information S1: Figure S10), underscoring the robustness of our proposed imaging mechanism.

### 2.4 Geometry Fidelity in Polaritonic Imaging

Finally, we examine the geometry fidelity of polaritonic imaging. For individual hBN slabs, the in‐plane isotropic nature of hyperbolic polaritons leads to circular IFCs (Figure 4a). Consequently, the object geometry is preserved in the emitted near‐field patterns, as demonstrated by the concentric interference rings with different light and shade originating from a gold disk antenna of radius *r*_Au_ = 4 μm. However, due to strong polaritonic resonances, the object's physical outline cannot be easily discerned from the interference‐dominated intensity patterns.

**FIGURE 4:**
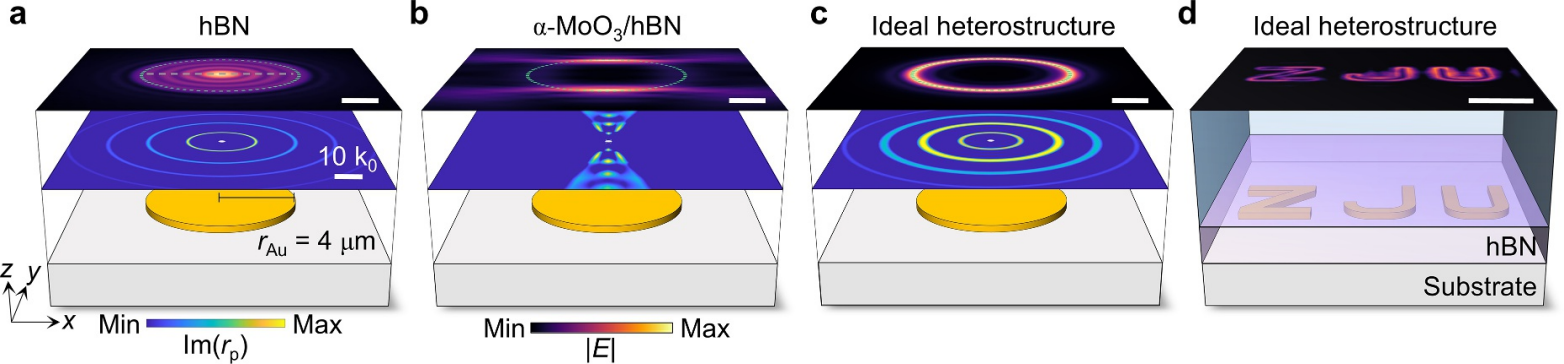
Geometry fidelity in polaritonic imaging. (a) Simulated field intensity distribution excited by a gold disc antenna with a radius of *r*_Au_ = 4 μm, overlaid on the calculated isofrequency contour. (b) Same as (a), but for an α‐MoO_3_/hBN heterostructure. (c) Same as (a), but for an ideal heterostructure obtained by replacing the dielectric permittivity of α‐MoO_3_ along the *x* direction with that along *y*. (d) Schematic illustration of the concept of high‐fidelity near‐field imaging for the “ZJU” letter antennas with the ideal heterostructure. Scale bars in top panels, 2 μm.

In contrast, when α‐MoO_3_ is integrated with hBN to form a vertical heterostructure, negative refraction significantly suppresses these resonances. The resulting near‐field intensity maxima align closely with the physical boundaries of the object and decay rapidly away from it (Figure 4b), thereby improving boundary recognition. Nevertheless, the image exhibits anisotropic distortion: the scale fidelity is preserved only along the *y* direction. This distortion primarily originates from the low‐symmetry IFCs involving in‐plane anisotropic α‐MoO_3_.

To overcome this limitation, we propose a design principle for achieving both scale and geometry fidelity: employing a polaritonic material with out‐of‐plane anisotropy resembling that of α‐MoO_3_ but with in‐plane isotropy. To verify this concept, we theoretically substitute the dielectric permittivity of α‐MoO_3_ along the *x* direction with that along *y* ($\varepsilon_{\text{Mo}O_{3}}^{x}\equiv \varepsilon_{\text{Mo}O_{3}}^{y}<0$) and preserve all the other parameters. This modification restores a circular IFC in Figure 4c. Correspondingly, the simulated near‐field image exhibits a circular halo that maintains both accurate scale and geometry, validating the feasibility of our design approach.

Beyond simple disc structures, we further evaluate the imaging performance of the proposed “ideal” heterostructure using objects with more complicated geometries, such as the letters “ZJU” shown in Figure 4d. The simulated field distribution faithfully reproduces the fine structural details with nearly 1:1 scale fidelity, demonstrating the potential of this approach for nonintrusive deep‐subwavelength imaging.

## 3 Conclusions

In summary, we unveil fundamental limits of polaritonic imaging by connecting its spatial resolution with the linewidths of hyperbolic polariton beams. We also propose FWHM‐based criterion and Sparrow criterion to evaluate its resolution limit and point out feasible approaches for enhancing the scale and geometry fidelities. As the resolving power is primarily governed by the loss of the system [39], techniques, such as isotope engineering [40, 41] and cryogenic imaging [42, 43, 44], that directly mitigate the intrinsic loss of polaritonic materials could be a promising direction for optimizing the performance of polaritonic imaging and satisfying the requirement in future nanometrology applications.

## 4 Methods

Full‐wave simulations were performed in COMSOL Multiphysics based on the finite element method. To enable direct comparison with previous work, the Cartesian axes (*x*, *y*, *z*) were aligned with the [100], [001], and [010] crystallographic directions of α‐MoO_3_, respectively. A two‐dimensional model with a minimum mesh size of 0.1 nm was built for field distribution simulations in the *y*–*z* plane, while a three‐dimensional model was constructed for the simulation in the *x*–*y* plane.

For structures composed of gold antennas, a point dipole was placed at the center of the antenna's bottom surface, while its top surface was set as a perfect electrical conductivity (PEC) boundary. Compared with plane‐wave excitation [20, 21], this configuration eliminates the impact of polarization directions, yielding symmetric field profiles reported in earlier experiments [43].

The dispersion relation shown in Figure 3b was calculated using the transfer matrix method [45], which was also used to calculate the IFCs in Figure 4a–c. The IFCs in Figure 3d were calculated by quasi‐analytically solving a waveguide‐like eigenfunction [46]. For simplicity, the surrounding dielectric environment was assumed to be air. The dielectric permittivities of hBN and α‐MoO_3_ were adopted from previously published values [29, 47].

## Author Contributions

**Tianran Yu:** methodology, software, writing – original draft. **Zhenyang Cui:** methodology. **Sihao Xia:** methodology. **Zhiwei He:** methodology. **Huaping Wang:** conceptualization. **Lian Shen:** conceptualization. **Hongsheng Chen:** supervision. **Yingjie Wu:** conceptualization, methodology, writing – review and editing, supervision.

## Conflicts of Interest

The authors declare no conflicts of interest.

## Supporting information


Supporting Information S1

## Data Availability

The data that supports the findings of this study are available in the supplementary material of this article.
