# Supplementary material for: Fundamentals of Deep‐Subwavelength Near‐Field Imaging With Hyperbolic Polaritons
Source: Nanophotonics. 2026 Apr 17;15(8):e70089. doi: 10.1002/nap2.70089 (PMC13642602; doi:10.1002/nap2.70089)
Supplement: Supplementary file 1 [file NAP2-15-e70089-s001.docx]

Supporting Information for

**Fundamentals of deep-subwavelength near-field imaging with hyperbolic polaritons**

Tianran Yu^1,2,3^, Zhenyang Cui^1,2,3^, Sihao Xia^1,2,3^, Zhiwei He^1,2,3^, Huaping Wang^1,2,3^, Lian Shen^1,2,3^, Hongsheng Chen^1,2,3*^, Yingjie Wu^1,2,3*^

^1^State Key Laboratory of Extreme Photonics and Instrumentation, ZJU-Hangzhou Global Scientific and Technological Innovation Center, Zhejiang University, Hangzhou, China.

^2^The Electromagnetics Academy at Zhejiang University, College of Information Science and Electronic Engineering, Zhejiang University, Hangzhou, China.

^3^Zhejiang Key Lab. of Intelligent Electromagnetic Control and Advanced Electronic Integration, Jinhua Institute of Zhejiang University, Zhejiang University, Jinhua, China.

^*^Correspondence to: [hansomchen@zju.edu.cn](mailto:hansomchen@zju.edu.cn) (H.C.), [yingjie.wu@zju.edu.cn](mailto:yingjie.wu@zju.edu.cn) (Y.W.).

**Note 1. Calculation of linewidths of hyperbolic polariton beams**

We begin by considering an electric line dipole source that extends infinitely along the *x* direction and is polarized along the *z* direction. Following refs. [1-3], in the cylindrical coordinate system $(d,\delta)$, the *z*-component of the magnetic vector potential ($A_{z}^{(0)}$) is given by

$A_{z}^{(0)}=\mu_{0}I\cdot\left[ \frac{i}{4}H_{0}^{\left( 1 \right)}\left( k_{0}d \right) \right]=\frac{\mu_{0}I}{4}\left( -i\omega p \right)H_{0}^{\left( 1 \right)}\left( k_{0}d \right)=\frac{\mu_{0}\omega p}{4}H_{0}^{\left( 1 \right)}\left( k_{0}d \right)$	(S1)

where $\mu_{0}$ is the vacuum permeability, $I=-i\omega p$ is the equivalent current of the line dipole, $\omega$ is the frequency (in rad/s), $p$ is the electric dipole moment, $H_{0}^{(1)}$ is the Hankel function of the first kind and order 0, and $k_{0}$ is the free-space wavevector. The magnetic field follows from

$\mathbf{H}^{(0)}=\frac{1}{\mu_{0}}\nabla\times A_{z}^{(0)}=\frac{1}{\mu_{0}}\left( \frac{\partial A_{z}^{(0)}}{\partial y}\hat{x}-\frac{\partial A_{z}^{(0)}}{\partial x}\hat{y} \right)$.	(S2)

Because the source is invariant along *x*, $A_{z}^{(0)}$ and hence $H_{x}^{(0)}$ are independent of $x$. Equation (S2) thus simplifies to

$H_{x}^{(0)}=\frac{1}{\mu_{0}}\frac{\partial A_{z}^{(0)}}{\partial y}=\frac{\omega p}{4}\frac{\partial}{\partial y}H_{0}^{\left( 1 \right)}\left( k_{0}d \right)$.	(S3)

Using the derivative identity for Hankel functions,

$\frac{d}{dd}H_{0}^{(1)}\left( k_{0}d \right)=-k_{0}H_{1}^{\left( 1 \right)}\left( k_{0}d \right)$,$\frac{\partial\rho}{\partial y}=\frac{y}{d}$,	(S4)

we rewrite the Equation (S3) as

$H_{x}^{(0)}=\frac{\omega p}{4}\frac{\partial}{\partial y}H_{0}^{\left( 1 \right)}\left( k_{0}d \right)=-\frac{\omega pk_{0}}{4}H_{1}^{\left( 1 \right)}\left( k_{0}d \right)\frac{y}{d}=-\frac{pck_{0}^{2}}{4}H_{1}^{(1)}\left( k_{0}d \right)\frac{y}{d}$,	(S5)

where $H_{1}^{(1)}$ is the Hankel function of the first kind and order 1 and $c$ is the speed of light. From Maxwell’s curl equation, $\nabla\times\mathbf{H}=i\omega\varepsilon_{0}\mathbf{E}$, the in-plane electric field components follow as:

$E_{y}^{(0)}=\frac{1}{i\omega\varepsilon_{0}}\frac{\partial H_{x}^{(0)}}{\partial z}$, $E_{z}^{(0)}=-\frac{1}{i\omega\varepsilon_{0}}\frac{\partial H_{x}^{(0)}}{\partial y}$,	(S6)

where $\varepsilon_{0}$ denotes the vacuum permittivity.

Equation S5 and Equation S6 describe the field of an infinite line dipole in free space. For hBN with opposite in-plane $(\varepsilon_{\mathrm{xy}})$ and out-of-plane ($\varepsilon_{z}$) permittivities, Clemmow’s scaling theorem provides a direct mapping between free-space and anisotropic solutions. In the *y*−*z* plane of hBN, energy propagates along a hyperbolic resonance cone whose angle $\theta$ relative to the *y*-axis is

$\theta=Re[atan\left( \sqrt{-\frac{\varepsilon_{z}}{\varepsilon_{\mathrm{xy}}}} \right)]$.	(S7)

The corresponding dispersion relation is

$\frac{k^{2}{cos\theta}^{2}}{\varepsilon_{z}}+\frac{k^{2}{sin\theta}^{2}}{\varepsilon_{\mathrm{xy}}}=\left( k_{0} \right)^{2}$.	(S8)

Solving for the wavevector $k$ gives

$k=\frac{k_{0}}{\sqrt{\frac{{cos\theta}^{2}}{\varepsilon_{z}}+\frac{{sin\theta}^{2}}{\varepsilon_{\mathrm{xy}}}}}=n_{\theta}k_{0}$,	(S9)

where $n_{\theta}$ is the effective refractive index associated with the resonance-cone direction. Clemmow’s scaling theorem maps the free-space electric fields into those in hBN as

$E_{y}=n_{\theta}E_{y}^{(0)}$, $E_{z}=n_{\theta}E_{z}^{(0)}$.	(S10)

The hyperbolic field intensity is thus

$\left| E \right|=\sqrt{E_{y}^{2}+E_{z}^{2}}$.	(S11)

The field distribution in α-MoO_3_ is obtained following a similar procedure. The linewidths of hyperbolic polariton beams in all cases are extracted as the FWHM from line-scan profiles taken perpendicular to the propagation direction.

**Note 2. Linewidth calculation in heterostructures**

The extracted linewidths in heterostructures (Figure S9) show nearly the same distance dependence as those in individual layers. The cross-sectional linewidth ($W_{c1}$) at the α-MoO_3_/hBN interface can be written as:

$W_{c1}=h_{\mathrm{hBN}}\left[ \cot\left( \theta_{\mathrm{hBN}}-\varphi_{\mathrm{hBN}} \right)-\cot\left( \theta_{\mathrm{hBN}}+\varphi_{\mathrm{hBN}} \right) \right]$,	(S12)

where $h_{\mathrm{hBN}}$ is the thickness of the hBN layer, and $\theta_{\mathrm{hBN}}$ and $\varphi_{\mathrm{hBN}}$ are the tilting and divergence angle of polariton beams in hBN.

The effective thickness of α-MoO_3_ ($h_{\mathrm{MoO}_{3}}^{\mathrm{eff}}$) that produces the same cross-sectional linewidth is given by:

$h_{\mathrm{MoO}_{3}}^{\mathrm{eff}}=\frac{W_{c1}}{\cot\left( \theta_{\mathrm{MoO}_{3}}-\varphi_{\mathrm{MoO}_{3}} \right)-\cot\left( \theta_{\mathrm{MoO}_{3}}+\varphi_{\mathrm{MoO}_{3}} \right)}$,	(S13)

where $\theta_{\mathrm{MoO}_{3}}$ and $\varphi_{\mathrm{MoO}_{3}}$ are the tilting and divergence angles in α-MoO_3_.

Substituting $h_{\mathrm{MoO}_{3}}^{\mathrm{eff}}$ into Equation (S12), the linewidth ($W_{c2}$) at the top surface of the α-MoO_3_ layer with the thickness of $h_{\mathrm{MoO}_{3}}$ can then be obtained as:

$W_{c2}=(h_{\mathrm{MoO}_{3}}^{\mathrm{eff}}+h_{\mathrm{MoO}_{3}})\left[ \cot\left( \theta_{\mathrm{MoO}_{3}}-\varphi_{\mathrm{MoO}_{3}} \right)-\cot\left( \theta_{\mathrm{MoO}_{3}}+\varphi_{\mathrm{MoO}_{3}} \right) \right]$.	(S14)

**Figure S1.** Electric field distributions obtained from numerical calculation (**a**) and full-wave simulation (**b**), corresponding to Figure 1a and Figure 1d in the main text, respectively. (**c**) Extracted line-scan profiles at *d* = 50 nm and 200 nm along the blue and red lines in **a** and **b**, where the intensity is normalized to the value at *d* = 50 nm.

**Figure S2.** Additional numerical calculations (**a**) and full-wave simulation results (**b**) for hyperbolic polariton beams in hBN at *ω* = 780, 800, and 810 cm^−1^. (**c**) The extracted linewidths agree well with the values calculated from the analytical model.

**Figure S3.** Calculation (**a**) and simulation results (**b**) of hyperbolic polariton beams excited by a point dipole polarized along the *y*-axis. (**c**) Calculated linewidth as a function of distance, in consistent with values extracted from **a** and **b**.

**Figure S4.** Hyperbolic polariton beams in α-MoO_3_. (**a**) Calculated electric field distribution in an α-MoO_3_ bulk crystal at *ω* = 790 cm^-1^. (**b**) Field intensity as a function of *d*. (**c**) Simulation result. (**d**) Relation between linewidth and radial distance.

**Figure S5.** (**a**) Full-wave simulations with gold antennas of different lengths. (**b**) Distance-dependent beam linewidths extracted from the simulations. The gray line is calculated based on the analytical model, without considering external loss.

**Figure S6.** (**a**) Simulated field distribution (top) and corresponding line-scan profile (bottom) in an hBN slab with *h*_hBN_ = 50 nm, excited by two laterally positioned dipoles with spacing *d*_dp._ = 5 nm, corresponding to the resolution limit defined by the FWHM criterion. (**b**) Same to **a** but with *d*_dp._ = 1 nm, corresponding to the resolution limit defined by the Sparrow criterion.

**Figure S7.** Simulation results corresponding to the heterostructure configuration in Figure 3a of the main text.

**Figure S8.** Additional simulation results for α-MoO_3_/hBN heterostructures at 780, 800, and 810 cm^−1^. The thickness of the hBN layer is fixed at 300 nm. To maintain scale fidelity, the corresponding thickness ratios $\frac{h_{\mathrm{MoO}_{3}}}{h_{\mathrm{hBN}}}$ are 1.21, 2.88, and 4.78, respectively.

**Figure S9.** (**a**) Simulated field distribution, corresponding to Figure 3c in the main text. (**b**) Extracted linewidths from **a**. (**c**) Schematic illustration of the linewidth calculation in the α-MoO_3_ layer.

**Figure S10.** (**a**) Near-field intensity profiles on the surfaces of an individual α-MoO_3_ slab (gray curves) and an hBN/α-MoO_3_ heterostructure (blue curves) placed on gold antennas with varying widths (*d*_Au_). (**b**) Dispersion in the hBN/α-MoO_3_ heterostructure. (**c**) Simulated field distribution in the hBN/α-MoO_3_ heterostructure.

**Table S1. Parameters for the calculation of dielectric permittivities.**

| Materials | $\varepsilon_{\infty}$ | $\omega_{\mathrm{LO}}$ (cm^−1^) | $\omega_{\mathrm{TO}}$ (cm^−1^) | $\gamma$ (cm^−1^) |
| --- | --- | --- | --- | --- |
| hBN, *xy* | 4.776 | 1630 | 1340 | 7 |
| hBN, *z* | 2.95 | 825 | 760 | 2 |
| α-MoO_3_, *x* | 4.8 | 974 | 824 | 6 |
| α-MoO_3_, *y* | 6.3 | 850 | 544.6 | 9.5 |
| α-MoO_3_, *z* | 2.2 | 1004 | 957 | 1.5 |

**References**

(1) Chew, W. C, *Waves and Fields in Inhomogeneous Media*, New York, USA: IEEE Press, 1995.

(2) Ishii, Satoshi, Alexander V. Kildishev, Evgenii Narimanov, Vladimir M. Shalaev, and Vladimir P. Drachev. “Sub‐wavelength Interference Pattern from Volume Plasmon Polaritons in a Hyperbolic Medium: Sub-Wavelength Interference in a Hyperbolic Medium.” *Laser & Photonics Reviews* 7, no. 2 (2013): 265–71. <https://doi.org/10.1002/lpor.201200095>.

(3) Potemkin, Andrey S., Alexander N. Poddubny, Pavel A. Belov, and Yuri S. Kivshar. “Green Function for Hyperbolic Media.” *Physical Review. A* 86, no. 2 (2012). <https://doi.org/10.1103/physreva.86.023848>.
